# Cysteine-Rich Protein 61 Plays a Proinflammatory Role in Obstructive Kidney Fibrosis

**DOI:** 10.1371/journal.pone.0056481

**Published:** 2013-02-15

**Authors:** Chun-Fu Lai, Yung-Ming Chen, Wen-Chih Chiang, Shuei-Liong Lin, Min-Liang Kuo, Tun-Jun Tsai

**Affiliations:** 1 Department of Internal Medicine, National Taiwan University Hospital and College of Medicine, Taipei, Taiwan; 2 Graduate Institute of Clinical Medicine, National Taiwan University College of Medicine, Taipei, Taiwan; 3 Graduate Institute of Physiology, National Taiwan University College of Medicine, Taipei, Taiwan; 4 Graduate Institute of Toxicology, National Taiwan University College of Medicine, Taipei, Taiwan; 5 Department of Internal Medicine, National Taiwan University Hospital Yun-Lin Branch, Douliou City, Taiwan; Universidade de Sao Paulo, Brazil

## Abstract

Cysteine-rich protein 61 (Cyr61) is a secreted matrix-associated protein that regulates a broad spectrum of biological and cellular activities. This study aimed to investigate the role of Cyr61 in progressive kidney fibrosis induced by unilateral ureteral obstruction (UUO) surgery in mice. The expression of Cyr61 transcripts and proteins in the obstructed kidneys were increased from day 1 and remained high until day 10 after surgery. Immunohistochemistry indicated that Cyr61 was expressed mainly in renal tubular epithelial cells. The upregulated Cyr61 in UUO kidneys was reduced in mice treated with pan-transforming growth factor-β (TGF-β) antibody. The role of TGF-β in tubular Cyr61 upregulation after obstructive kidney injury was further supported by experiments showing that TGF-β1 stimulated Cyr61 expression in cultured tubular epithelial cells. Notably, the upregulation of Cyr61 in UUO kidneys was followed by a marked increase in monocyte chemoattractant protein 1 (*MCP-1*) transcripts and macrophage infiltration, which were attenuated in mice treated with anti-Cyr61 antibodies. This proinflammatory property of Cyr61 in inducing *MCP-1* expression was further confirmed in tubular epithelial cells cultured with Cyr61 protein. The anti-Cyr61 antibody in UUO mice also reduced the levels of collagen type 1-α1 transcripts, collagen fibril accumulation evaluated by picrosirius red staining, and the levels of α-smooth muscle actin (*α-SMA*) transcripts and proteins on day 4 after surgery; however, the antifibrotic effect was not sustained. In conclusion, the TGF-β-mediated increase in tubular Cyr61 expression involved renal inflammatory cell infiltration through MCP-1 induction during obstructive kidney injury. The Cyr61 blockade attenuated kidney fibrosis in the early phase, but the antifibrotic effect could not be sustained.

## Introduction

Chronic tubulointerstitial fibrosis is regarded as the final common pathway leading to end-stage renal failure [1]–[3]. During the evolution of renal fibrosis, multiple pathologic mechanisms occur, including inflammation, proteolysis, hypoxia, tubular decomposition, and extracellular matrix accumulation [4]. So far, there is no effective therapeutic strategy capable of fully stopping its progression. Therefore, the identification of the detailed mechanisms of tubulointerstitial fibrosis with the ultimate goal of halting renal disease progression is of great interest to researchers.

Cysteine-rich protein 61 (Cyr61) is a secreted, matrix-associated, heparin-binding protein belonging to the “CCN” family, which comprises Cyr61 (CCN1), connective tissue growth factor (CTGF, CCN2), nephroblastoma overexpressed (NOV, CCN3), WNT 1-inducible signaling pathway proteins (WISP)-1 (CCN4), WISP-2 (CCN5), and WISP-3 (CCN6) [5]–[7]. Cyr61 has been reported to control the cell cycle, stimulate chemostasis, and augment growth factor-induced effects [6], [8]. It also participates in angiogenesis by promoting endothelial cell survival and stimulating pro-angiogenic factors [9]–[11]. In addition, Cyr61 has been shown to integrate the biological mechanisms of cutaneous wound healing. It can regulate the expression of genes involved in matrix remodeling [9] and induce senescence and apoptosis in fibroblasts [11], [12]. Recently, it was discovered that Cyr61 promoted inflammation and modified the effects of cytokines on cell death [6], [13], [14]. All of these mechanisms in which Cyr61 is involved are crucial processes in the context of renal fibrosis progression [4].

Animal and human studies have demonstrated Cyr61 expression in normal and diseased kidneys [15]–[17]. Cyr61 shares structural and functional similarity with CTGF [5], [7]. Both of theses proteins can be regulated by transforming growth factor-β (TGF-β) [5]. There is increasing evidence suggesting that TGF-β and CTGF participate in the pathophysiology of chronic tubulointerstitial fibrosis [3], [18]–[21]. However, the role of Cyr61 in renal fibrosis remains largely unknown. Hence, we hypothesized that Cyr61 plays a functional role in the pathogenesis of renal tubulointerstitial fibrosis. By inducing unilateral ureteral obstruction (UUO) in mice, we investigated Cyr61 expression in this progressive renal fibrosis model. Our results indicated that Cyr61 contributes to the inflammatory process in chronic kidney injury. Cyr61 blockade attenuated kidney fibrosis in the early phase, but the antifibrotic effect could not be sustained.

## Materials and Methods

### Reagents

Antibodies against Ser423/425-phosphorylated Smad2/3 (pSmad2/3) and GAPDH were from Santa Cruz Biotechnology (CA, USA), while antibodies against total Smad2/3 (tSmad2/3) were from Cell Signaling Technology (MA, USA). Normal rabbit IgG and anti-α-smooth muscle actin (*α-SMA*) antibodies were purchased from Sigma (MO, USA). The pan-TGF-β monoclonal antibody (1D11) and its control antibody (13C4) were kind gifts from Dr. Hong Ling (Genzyme Corp, Cambridge, MA, USA) [22]. Recombinant TGF-β1 protein was from R&D Systems (Minneapolis, MN, USA), while recombinant Cyr61 and CTGF protein were from PeproTec (Rocky Hill, NJ, USA). DMEM medium, fetal bovine serum (FBS), and other cell culture reagents were obtained from Gibco BRL (Rockville, MD, USA).

The sequence CSKTKKSPEPVRFTY (residue #284-298) was selected from the mouse *Cyr61* gene (GenBank AAH66019.1) by maximizing hydrophilicity, antigenicity, surface probability, and excluding regions that do not contain turns or that contain glycosylation sites. Peptides synthesized using this sequence were produced chemically and coupled with the carrier protein ovalbumin. Two rabbits were immunized with this synthesized peptide by intramuscular injection at a ten-week interval (6 injections of 0.5 mg/rabbit/injection). After the sixth immunization, the antiserum was purified by affinity chromatography (protein A agarose resins, ABT, Tampa, FL). The column was washed extensively with PBS, and the antibodies were eluted with 100 mM glycine, pH 3.0. This rabbit anti-mouse Cyr61 polyclonal antibody was used as a neutralizing antibody and for immunohistochemistry and Western blotting.

Normal rat kidney proximal tubular epithelial cells (NRK-52E) and fibroblast cells (RNK-49F) were cultured as stated previously [23]. The cells were cultured in DMEM medium supplemented with 10% FBS. Subconfluent cells were made quiescent by placing them in medium with 0.1% FBS for 16 hours before the experiments.

### Animal model

Eight-week-old adult male ICR mice, weighing 25–30 g, were obtained from the laboratory animal center of the National Taiwan University College of Medicine. Under anesthesia, UUO was induced by ligation of the left ureter as described previously [24]. UUO mice were euthanized at 1, 2, 4, 7, or 10 days after surgery (N = 8 at each time point). The kidneys were removed and divided into parts. As previously described [19], a part of the kidney was fixed in 10% neutral buffered formalin, another part was fixed in 4% paraformaldehyde/PBS at 4°C, and the remainder was snap frozen in liquid nitrogen and stored at −70°C. Age-matched, sham-operated mice had undergone left ureter manipulation but not ligation. These sham-operated mice were euthanized one day after the operation; their kidneys served as controls (N = 8). In some experiments, the mice were treated with 10 µg/g body weight (BW) of pan-TGF-β monoclonal antibody (1D11) [22] or its control antibody (13C4) intraperitoneally 2 hours before UUO surgery and were euthanized the next day (N = 4 for each group). In other experiments, 10 µg/g BW of anti-Cyr61 antibody or control IgG were injected intraperitoneally 2 hours before UUO surgery, and then one dose was injected per day until the animals were euthanized (N = 8 for each group). All of the animal studies were performed under a protocol approved by the Institutional Animal Care and Use Committee, National Taiwan University College of Medicine. All of the surgeries were performed under sodium pentobarbital anesthesia, and all efforts were made to minimize the animals' suffering.

### RNA Extraction and Quantitative PCR (Q-PCR)

Total tissue or cell RNA was isolated using TRIzol reagent (Invitrogen, Carlsbad, CA). Then, cDNA was synthesized by using the iScript cDNA Synthesis kit (Bio-Rad Lab., Hercules, CA, USA) following the manufacturer's instructions. Q-PCR was performed using methods described previously [25]. The specific primer pairs used for PCR are listed in Table 1.

**Table 1:** Primer sequences used for quantitative-PCR.

Primer	Forward	Backward
Cyr61	5′-ATTCTTGAGTAGCATTAGG-3′	5′-GTACTATGAAGCGAAGTC-3′
CTGF	5′-GACACGAACTCATTAGACTAT-3′	5′-GAGGTTGACAGACTACTTG-3′
MCP-1	5′-GTGAAGTTGACCCGTAAATC-3′	5′-CTCCTACAGAAGTGCTTGA-3′
Col 1-α1	5′-ACGAACAACCCAAACTCA-3′	5′-GTTCAGTTGGTCAAAGGTAAA-3′
TGF-β1	5′-AGACATCTCACACAGTAT-3′	5′-CCAGGAATTGTTGCTATA-3′
CCR-2	5′-ATTCTCCACACCCTGTTTCG-3′	5′-GATTCCTGGAAGGTGGTCAA-3′
F4/80	5′-CCTGGTGGTCATAATCTC-3′	5′-GGAGGACAGAGTTTATCG-3′
CCL17	5′-AGTGGAGTGTTCCAGGGATG-3′	5′-CTGGTCACAGGCCGTTTTAT-3′
CCL22	5′-TGGCTACCCTGCGTGTCCCA-3′	5′-GCCAGGCTTGCGGCAGGATT-3′
α-SMA	5′-GACGCTGCTCCAGCTATGTG -3′	5′-CAGCGTCAGGATCCCTCTCT-3′
18S	5′-GTTGGTTTTCGGAACTGAGGC -3′	5′-GTCGGCATCGTTTATGGTCG-3′

### Protein Extraction and Western Blot Analyses

Total protein from the kidney tissue or cell extracts were dissolved using RIPA buffer and were subjected to Western blot analysis using previously stated methods [26]. The blots were incubated with primary antibodies at 4°C overnight, further incubated with peroxidase-conjugated secondary antibodies, and then visualized using chemiluminescence reagents (Millipore, MA, USA) according to the manufacturer's instructions.

### Immunoassay of TGF-β1

For tissue TGF-β1 concentrations measurement, kidneys were homogenized in PBS containing protease inhibitor (Thermo, Rockford, IL, USA). The homogenates were centrifuged at 10,000 *g* for 30 minutes at 4°C. The supernatants were recovered for active TGF-β1 levels determination by using an immunoassay kit (Quantikine, R&D system, Minneapolis, MN, USA). To activate latent TGF-β1 to the immunoreactive form, samples were acidified and then neutralized before measurements for the total TGF-β1 assay, according to the manufacturer's instructions. Values were expressed as pg/mg protein for the protein extract.

### Histological Examination and Immunohistochemistry

For immunohistochemical staining, deparaffinized kidney sections were subsequently treated with proteinase K (Promega) and microwave heating in Epitope Retrieval Solution (Leica, UK). The sections were immersed in 3% H_2_O_2_ to block the endogenous peroxidase and then in Protein Block (Leica, UK) to block non-specific binding sites. After overnight incubation with anti-Cyr61 antibody (1 ∶ 200) at 4°C, the sections were incubated in NovoLink Polymer (Leica, UK) for 30 minutes. The reactions were detected with peroxidase substrate containing diaminobenzidine (DAB) chromogen. Anti-Cyr61 antibody was omitted in the sections that served as negative controls.

Snap-frozen fresh kidney was prepared and cut as cryostat sections for immunofluorescence staining. A primary antibody against the F4/80 marker (1∶200, Invitrogen, Carlsbad, CA, USA) was used for immunolabeling. Fluorescent-conjugated, anti-rat FITC, secondary antibody (1∶250, Jackson Immunoresearch, PA, USA) labeling, co-labeling with 4′,6-diamidino-2-phenylindole (DAPI), and mounting with Vectashield were performed as previously described [24]. Images were captured and processed by confocal microscope (LSM 510 META, Carl Zeiss, Jena, Germany). To study the renal collagen matrix distribution, picrosirius red staining of paraffin sections was performed by established methods [24]. The sections were assessed morphometrically by using Fovea Pro 4.0 software (Reindeer Graphics, Asheville, NC, USA) to quantify the area of tissue occupied by positive staining for F4/80 or picrosirius red [27]. At least 10 randomly selected cortical interstitial field images were taken, and the mean area was calculated for each animal.

### Statistical Analysis

The results are expressed as the mean+standard deviation (SD). The statistical analyses were evaluated by a nonparametric Mann–Whitney *U*-test and performed using Stata 10.0 (StataCorp, College Station, TX, USA). A P value <0.05 was considered to be statistically significant.

## Results

### Increased renal Cyr61 expression following UUO surgery

**Figure 1:**
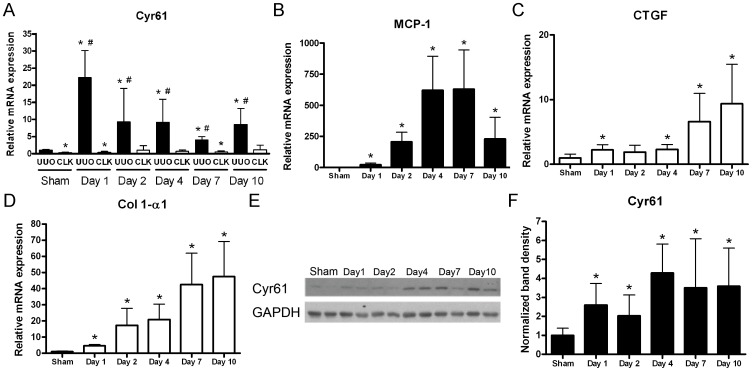
Increased Cyr61 expression in the kidneys after unilateral ureteral obstruction (UUO) surgery. (**A–D**) The Q-PCR time-course for transcripts of whole kidney *Cyr61* (A), monocyte chemoattractant protein-1 (*MCP-1*) (B), connective tissue growth factor (*CTGF*) (C), and collagen type I -α1 (*Co l 1-α1*) (D) following UUO surgery. The expression levels were normalized by 18S ribosomal RNA. N = 8/time point. (**E**) Representative images of the time-course Western blot of whole kidney lysates for Cyr61 after surgery. Glyceraldehyde 3-phosphate dehydrogenase (GAPDH) was used as a loading control. (**F**) Bar chart showing the Cyr61 protein expression time-course normalized by GAPDH. N = 8/time point. The values are the mean+SD. *P<0.05 vs. sham operation; #P<0.05 vs. contralateral (CLK) kidney.

As shown in Figure 1A, *Cyr61* transcripts in the UUO kidneys increased dramatically 1 day after surgery and remained increased at the subsequent time points until day 10. In the contralateral non-obstructed kidney, the *Cyr61* transcripts were not upregulated. Comparable to the increased transcripts, Cyr61 protein expression was already increased in the UUO kidneys from day 1 after the surgery, and the increased expression levels remained at subsequent time points (Figure 1E and F). The transcripts of monocyte chemoattractant protein-1 (*MCP-1*) increased markedly from day 2 and peaked at day 7 (Figure 1B). In contrast to the marked increase in *Cyr61* transcripts at day 1 after surgery, *CTGF* gene expression in the UUO kidneys increased markedly after day 7 following surgery but in a lesser degree in early time points (Figure 1C). Accompanying the increased *CTGF* expression was an increase in the transcript levels of collagen type I α-1 (*Col 1-α1*) in the UUO kidneys (Figure 1D). These findings suggest a possible link between CCN protein and renal inflammation and fibrosis in progressive obstructive kidney disease.

### Increased Cyr61 expression in tubular epithelial cells

**Figure 2:**
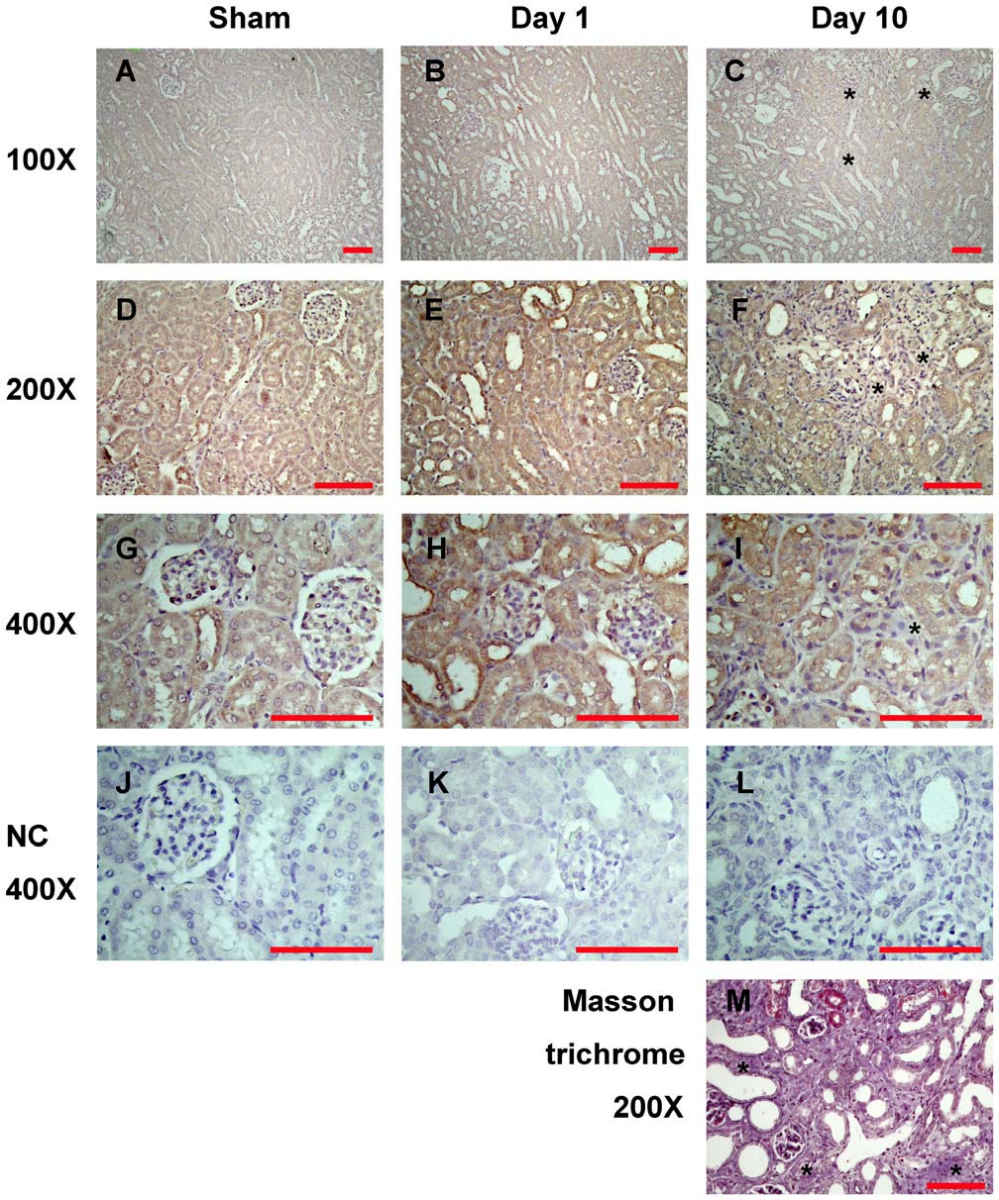
Localization of Cyr61 expression in the UUO kidneys. Representative immunohistochemical staining for Cyr61 protein in the sham operation kidneys (A, D, G, J), and the UUO kidneys at day 1 (B, E, H, K) and day 10 (C, F, I, L) after surgery. Low-power field images (A–F) show the major distribution of Cyr61 in renal tubular epithelial cells. Images of corticomedullary junctions (A–C) disclose that Cyr61 staining was positive in all renal tubule segments within the renal cortex and medulla. High-power field images (G–I) reveal that Cyr61 expression in the tubular epithelial cells was prominent in the cytoplasm. The star marks (*) indicate the lack of significant positive stain in the fibrotic area. Negative control (NC) staining showed no signal (J–L). (M) Masson trichrome stain of the corresponding day 10 UUO kidney shows the blue staining of collagen. Scale bar = 100 µm.

Cyr61 was detected by immunohistochemistry mainly in renal tubular epithelial cells in the sham operation and obstructed kidneys (Figure 2). Compatible with the previous report [17], Cyr61 staining was positive in all renal tubule segments within the renal cortex and medulla (Figure 2A–F). High-power observation revealed its expression in the tubular cells was prominent in the cytoplasm (Figure 2G–I). In later stages of UUO kidneys (day 10), interstitial fibrosis was apparent (Figure 2M); but there was a lack of significant Cyr61 staining in the fibrotic area (Figure 2C, F and I).

### TGF-β upregulated Cyr61 in UUO kidneys

**Figure 3:**
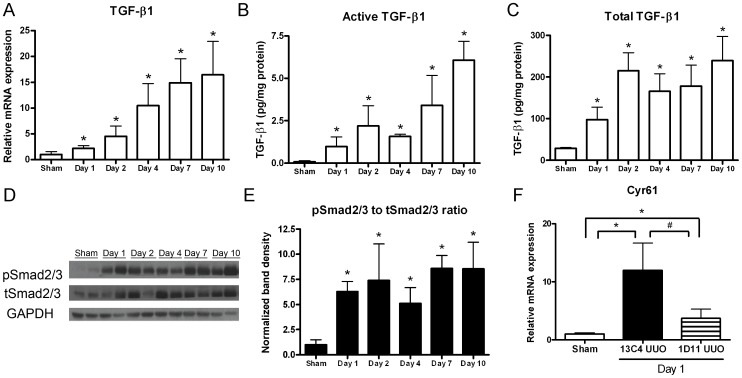
Transforming growth factor-β (TGF-β) activity is associated with *Cyr61* gene expression after UUO. (**A**) Expression of *TGF-β1* transcripts normalized to 18S in the UUO kidneys, measured by Q-PCR. N = 8/time point. (**B–C**) Active (B) and total (C) TGF-β1 protein concentrations as determined by immunoassay in kidneys of mice receiving sham operation or UUO surgery. N = 4/time point. (**D**) Representative Western blot of Smad2/3 proteins in the UUO kidneys. (**E**) The graph summarizes the analysis for renal phosphorylated Smad2/3 normalized to total Smad2/3 after UUO. N = 4/time point. (**F**) The mice received an intraperitoneal injection of 10 µg/g BW pan-TGF-β monoclonal antibody (1D11) or control antibody (13C4) 2 hours before the UUO surgery and were euthanized the next day. Q-PCR showed that the increased *Cyr61* transcripts in the UUO kidneys were attenuated by 1D11 treatment. N = 4/group. The values are the mean+SD. *P<0.05 vs. sham operation kidneys; #P<0.05 vs. 1D11 treated.

Previous evidence has suggested that TGF-β is a regulator of Cyr61 [5], [6], [8], [28]. TGF-β is also involved in the pathogenesis of kidney fibrosis [29], [30]. In our study, basal *TGF-β1* gene expression was low in the sham operation kidneys. After ureteral obstruction, *TGF-β1* transcripts in the kidney increased from day 1 and further elevated gradually (Figure 3A). It is known that the majority of TGF-β protein is stored in the extracellular matrix in the latent form, which could be activated into mature active form in response to stimulation [20]. The concentrations of active and total TGF-β1 in extracts of day 1 UUO kidneys were 14.2- and 3.4-fold higher than the sham operation kidneys, respectively (Figure 3B and C). These elevations became more prominent at the subsequent time points. To clarify the changes of activated TGF-β signaling in the UUO kidneys, we further measured the phosphorylation of Smad2/3, the major effectors of TGF-β signaling pathway. As shown in Figure 3D and E, Smad2/3 phosphorylation was markedly increased since day 1 and persisted until day 10 after UUO surgery.

To study whether TGF-β signaling regulates Cyr61 expression in UUO kidneys, TGF-β signaling was blocked in mice with a pan-TGF-β antibody. Q-PCR showed increased *Cyr61* transcript levels in day 1 UUO kidneys from control antibody (13C4)-treated mice. This increase was attenuated in pan-TGF-β antibody (1D11)-treated mice (Figure 3F). These findings indicate the involvement of TGF-β signaling in the upregulation of Cyr61 during obstructive kidney injury.

### Cyr61 expression in cultured renal tubular epithelial cells

**Figure 4:**
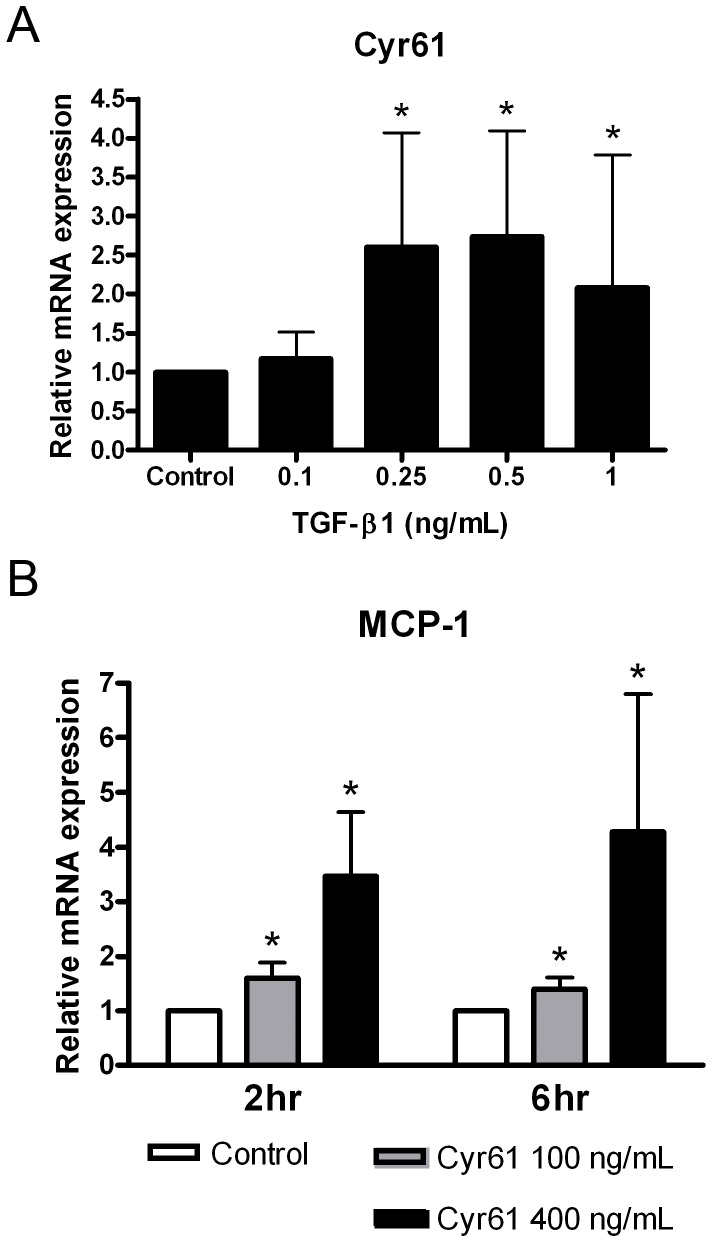
Cyr61 expression in cultured renal tubular epithelial cells. (**A**) Cultured rat proximal tubular epithelial cells (NRK-52E) were treated with various concentrations of recombinant TGF-β1 for 2 hours. Q-PCR showed *Cyr61* upregulation by TGF-β1 at doses of 0.25 ng/mL or greater. N = 4/group. (**B**) Recombinant Cyr61 was added to NRK-52E cells for 2 or 6 hours. Q-PCR showed increased expression of *MCP-1* transcripts by Cyr61 in a dose-dependent manner. N = 4/group. The values are the mean+SD. *P<0.05 vs. control.

To explore the role of TGF-β in Cyr61 expression in renal tubular epithelial cells, we performed TGF-β stimulation *in vitro*. Recombinant TGF-β1 at doses of 0.25 ng/mL or greater could increase *Cyr61* transcripts in NRK-52E cells (Figure 4A). Because Cyr61 was upregulated before significant MCP-1 in UUO kidneys in the time-course study (Figure 1), we were intrigued by the possible effect of Cyr61 on MCP-1 expression in cultured tubular epithelial cells. Recombinant Cyr61 induced *MCP-1* expression in NRK-52E cells in a dose-dependent manner (Figure 4B). These findings indicated that Cyr61 is a proinflammatory molecule in renal tubular epithelial cells.

### Validation of the anti-Cyr61 antibody

**Figure 5:**
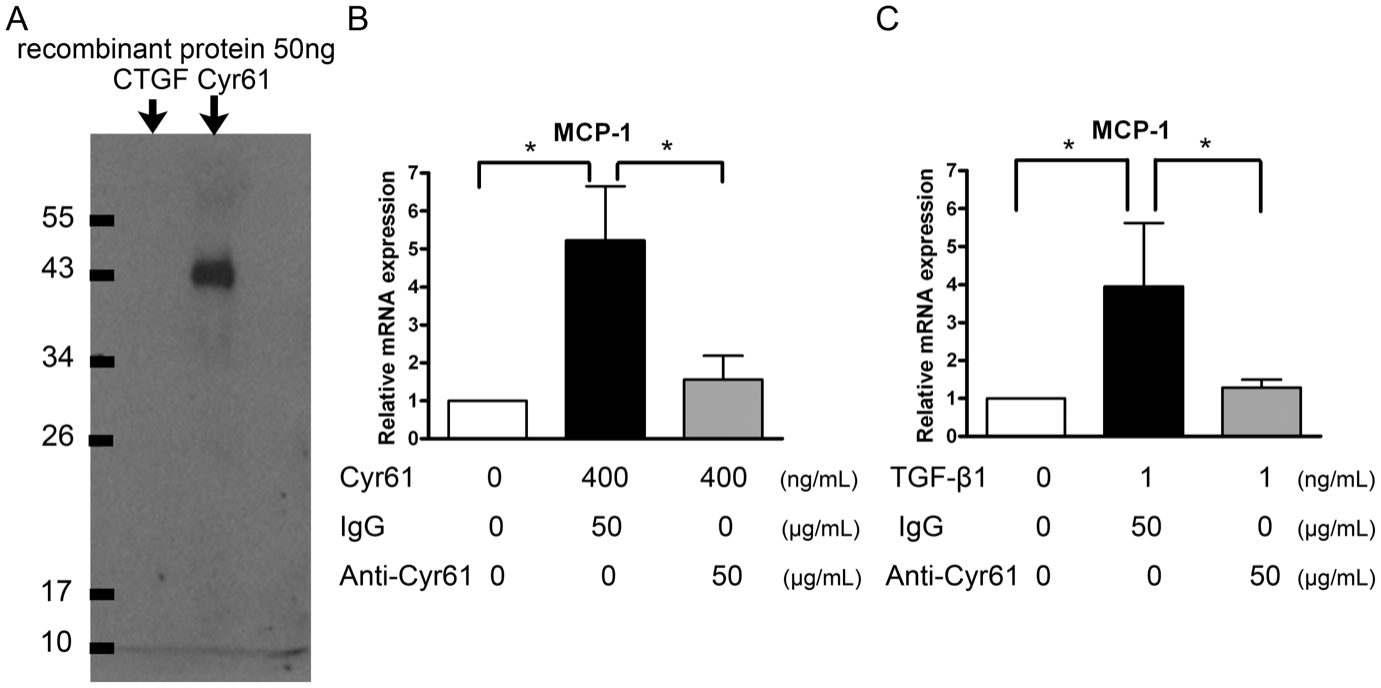
Structure specificity and neutralizing activity of anti-Cyr61 antibody. (**A**) Western blot of 50 ng recombinant CTGF and Cyr61 protein detected by an anti-Cyr61 antibody. This anti-Cyr61 antibody could specifically recognize Cyr61 but not structurally relevant CTGF. (**B**) Culture medium containing 400 ng/mL of recombinant Cyr61, pretreated with 50 µg/mL anti-Cyr61 antibody or control rabbit IgG for 1 hour, were added to NRK-52E cells for 2 hours. Q-PCR showed a significant reduction in Cyr61-enhanced *MCP-1* expression after anti-Cyr61 antibody pretreatment. N = 4/group. (**C**) The NRK-52E cells were treated with 1 ng/mL of TGF-β1 and 50 µg/mL of anti-Cyr61 antibody or control rabbit IgG for 24 hours. Q-PCR showed that anti-Cyr61 antibody attenuated TGF-β1-induced *MCP-1* upregulation. N = 4/group. The values are the mean+SD. *P<0.05.

To further investigate the effects of Cyr61, we produced anti-mouse Cyr61 polyclonal antibody by immunizing rabbits with the synthesized peptide selected from the *Cyr61* gene sequence. This anti-Cyr61 antibody was specific for Cyr61 but not structurally relevant CTGF, as shown by Western blot analysis (Figure 5A). Compared to the control rabbit IgG, the anti-Cyr61 antibody attenuated *MCP-1* expression in Cyr61-stimulated NRK-52E cells (Figure 5B). This finding indicates the structure specificity and neutralizing activity of this anti-Cyr61 antibody. (Also see Supplement Figure S1).

TGF-β1 had been shown to increase MCP-1 expression in renal tubular epithelial cells [31]. We subsequently treated NRK-52E cells with TGF-β1 with either control rabbit IgG or anti-Cyr61 antibody for 24 hours. The anti-Cyr61 antibody could attenuate TGF-β1-induced *MCP-1* upregulation (Figure 5C). These data provide evidence supporting the TGF-β→Cyr61→MCP-1 axis.

### Cyr61 antagonism reduced MCP-1 expression in UUO kidneys

**Figure 6:**
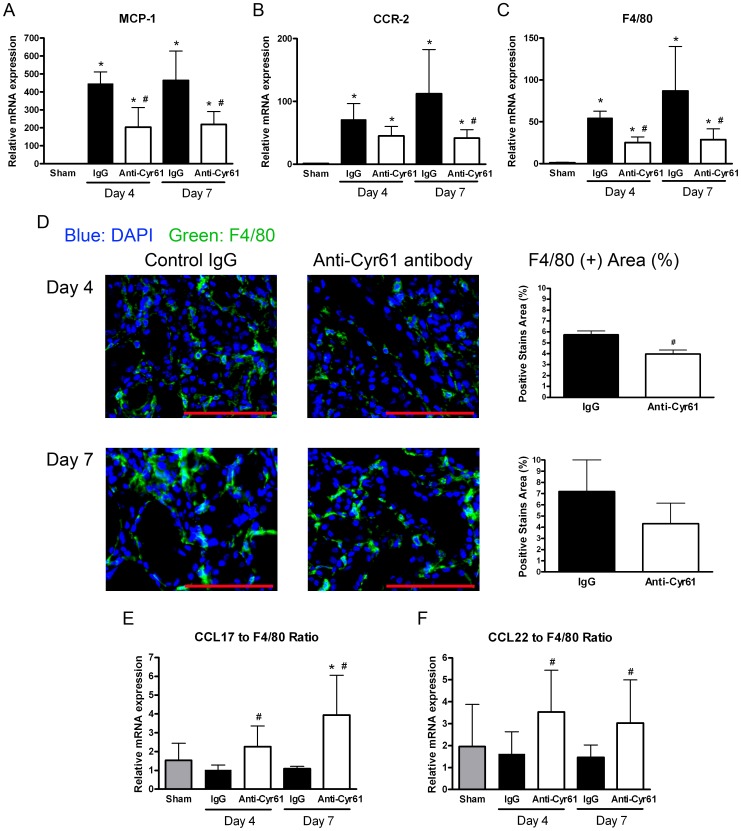
Effect of Cyr61 blockade on renal inflammation in the UUO kidneys. The mice received an intraperitoneal injection of 10 µg/g BW of the control rabbit IgG (filled bar) or anti-Cyr61 antibody (open bar) 2 hours before the UUO surgery and then one dose per day until euthanasia. (**A–C**) Graphs showing a summary of *MCP-1* (A), chemokine (C-C motif) receptor-2 (*CCR-2*) (B), and *F4/80* (C) transcripts normalized to *18S*. The data are expressed as the fold differences compared to the sham operation kidneys (gray bar). N = 8/group. (**D**) Representative F4/80 immunofluorescence photomicrographs (magnification 400×, scale bar = 100 µm) of kidneys from mice at days 4 and 7 after UUO. The bar charts are a summary of the percentage of positive F4/80-stained areas. N = 4/group. (**E and F**) Quantifying the expression of the profibrogenic macrophage-associated chemokine (C-C motif) ligand 17 (*CCL17*) and *CCL22* transcripts by Q-PCR. Graph showing the ratios of *CCL17* to *F4/80* (D) and *CCL22* to *F4/80* (E). All of the values are the mean+SD; *P<0.05 vs. sham operation; #P<0.05 vs. control IgG; N = 8/group.

To clarify the functional role of Cyr61 in progressive renal fibrosis, we examined the effect of Cyr61 blockade in a mouse UUO model. Compared to the sham operation kidneys, *MCP-1* transcripts in the UUO kidneys increased by 447- and 464-fold at days 4 and 7 after surgery, respectively, in mice treated with control IgG. Anti-Cyr61 antibody treatment reduced the upregulation of *MCP-1* transcripts in the UUO kidneys by 54.3 and 52.8% at days 4 and 7, respectively (Figure 6A). The functional relevance of renal *MCP-1* downregulation by Cyr61 antagonism was corroborated by the decreased transcription of the MCP-1 receptor, chemokine (C-C motif) receptor 2 (*CCR2*), and macrophage-/monocyte-specific cell surface marker *F4/80* (Figure 6B and C). Treatment with anti-Cyr61 antibody led to a significant reduction in F4/80-positive macrophages infiltration into UUO kidneys compared to treatment with control IgG at day 4 after surgery but not at day 7 (Figure 6D).

### Effect of Cyr61 antagonism on fibrosis in mouse UUO kidneys

**Figure 7:**
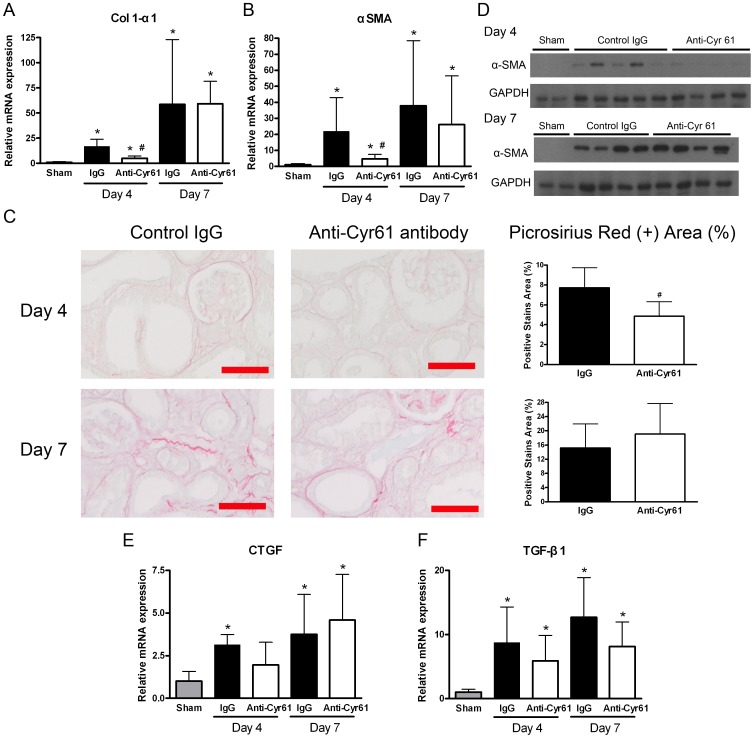
Effect of Cyr61 blockade on renal fibrosis in UUO kidneys. The mice received an intraperitoneal injection of 10 µg/g BW of the control rabbit IgG (filled bar) or anti-Cyr61 antibody (open bar) 2 hours before the UUO surgery and then one dose per day until euthanasia. (**A–B**) Q-PCR showing renal *Col 1-α1* (A) and α-smooth muscle actin (*α-SMA*) (B) transcripts after UUO. N = 8/group. (**C**) Representative images of picrosirius red staining for interstitial fibrillar collagen (red) in the UUO kidneys (magnification 200×, scale bar = 100 µm). The bar charts are a summary of the picrosirius red-stained percentage in the field. N = 8/group. (**D**) Representative images of Western blots of renal α-SMA protein expression in the UUO kidneys. (**E–F**) Q-PCR showing no difference in renal *CTGF* (E) and *TGF-β1* (F) transcripts between the 2 groups. N = 8/group. All of the Q-PCR data are expressed as the fold differences compared to the sham operation kidneys (gray bar). The values are the mean+SD. *P<0.05 vs. sham operation; #P<0.05 vs. control IgG.

Increased levels of the *Col 1-α1*, *CTGF*, and *TGF-β1* transcripts were observed in the UUO kidneys (Figure 1C and D; Figure 3A; Figure 7A, E, and F). Compared to the mice treated with control IgG, the anti-Cyr61 antibody treatment reduced *Col 1-α1* in the UUO kidneys by 69.5% at day 4 after surgery (Figure 7A). This inhibitory effect of Cyr61 antagonism on *Col 1-α1* was further shown by decreased picrosirius red staining for collagen fibrils in day 4 UUO kidneys (Figure 7C). However, this antifibrotic effect by Cyr61 antagonism was not observed in day 7 UUO kidneys (Figure 7A and C). Similarly, *α-SMA* RNA and protein overexpression in UUO kidneys was attenuated in anti-Cyr61 antibody-treated mice at day 4 after surgery but not at day 7 (Figure 7B and D). In addition, there was no discernible effect on *CTGF* and *TGF-β1* in comparisons of the mice treated with control IgG and the mice treated with anti-Cyr61 antibody (Figure 7E and F).

To determine why the early anti-inflammatory and anti-fibrotic effect of Cyr61 antagonism no longer translated to the later time points in the animal model of UUO kidney fibrosis, we studied macrophage polarization. Chemokine (C-C motif) ligand 17 (CCL17) and CCL22 are two markers of macrophages with a profibrotic phenotype [25], [32]–[34]. The transcript levels of total *CCL17* and *CCL22* in day 4 and 7 UUO kidneys were not different between control IgG-treated mice and anti-Cyr61 antibody-treated mice (data not shown). Considering the decreased *F4/80* expression in mice treated with anti-Cyr61 antibody, the ratios of *CCL17* to *F4/80* and *CCL22* to *F4/80* were used to assess the relative proportion of macrophages with profibrotic phenotype. Significantly higher ratios of *CCL17* to *F4/80* and *CCL22* to *F4/80* in the mice treated with anti-Cyr61 antibody were observed at days 4 and 7 after UUO surgery (Figure 6E and F). These findings indicate that Cyr61 might not only induce monocyte/macrophage infiltration through MCP-1 but may also polarize macrophages toward a proinflammatory phenotype rather than a profibrotic phenotype.

In addition to the proinflammatory effect on macrophage polarization, we further examined the effect of Cyr61 on kidney fibroblasts. Recombinant Cyr61 was found to downregulate the transcripts of *Col 1-α1* and *Col 3-α1* in cultured NRK-49F cells (Supplement Figure S2). These findings suggest that continuous Cyr61 blockade might lead to undesirable profibrotic effects on kidney fibroblast by increasing collagen transcription.

## Discussion

This study demonstrated that, through TGF-β stimulation, there was an early and continuous upregulation of Cyr61 in renal tubular epithelial cells during the course of kidney fibrosis after complete ureteral obstruction. In addition, Cyr61 played a proinflammatory role in the disease progression by inducing MCP-1 expression. Cyr61 blockade attenuated renal inflammation and ameliorated the severity of fibrosis in the early phase. However, this effect did not persist to later periods and could not translate into a lasting improvement on renal fibrosis.

TGF-β is a well-known key factor in the development of kidney fibrosis [21], [35]. It stimulates a variety of profibrotic factors, induces fibroblast proliferation, and has chemoattractive effects on immune cells [20], [21]. Treatment with anti-TGF-β antibody has been shown to ameliorate renal fibrosis in the UUO kidneys [36], [37]. However, long-term inhibition of TGF-β has been shown to cause a paradoxically worse renal outcome, possibly due to halting its anti-inflammatory properties [20], [38]. Isaka *et al.* showed that although renal fibrosis could be attenuated by TGF-β1 antisense nucleotide treatment in the UUO kidneys, macrophage infiltration in the obstructed kidneys did not change [39]. Therefore, it was suggested that therapeutic strategies should be explored, targeting the downstream effectors or the specific TGF-β signaling pathways [3]. One example of such a target for modulating the profibrotic activity of TGF-β is CTGF [3], [20], [24]. Our previous animal studies demonstrated the renoprotective effect of pentoxifylline by reducing the proliferation and extracellular matrix production of fibroblasts through the inhibition of CTGF [19], [23]. In this study, we reconfirmed the presence of CTGF overexpression during UUO. It became more prominent at days 4–7 of the disease, which was compatible with the appearance of significant renal fibrosis. However, Cyr61 upregulation started very early and might participate in different pathogenesis processes during progressive renal fibrosis compared to CTGF.

The similar timing of elevated tissue TGF-β1 concentration, enhanced Smad phosphorylation, and Cyr61 upregulation in UUO kidneys implied their association. This was supported by another report showing a classical Smad-binding motif in the Cyr61 promoter [28]. It should be emphasized that Smad activation is not the same as TGF-β activity because other factors, such as angiotensin II, can phosphorylate Smads without inducing TGF-β [35], [40]. However, we have shown suppression of Cyr61 upregulation using a pan-TGF-β antibody *in vivo*. Our *in vitro* experiments on renal tubular epithelial cells also supported this finding. Therefore, we inferred that TGF-β can regulate Cyr61 expression in UUO kidneys. Cyr61 may be another potential target to modify TGF-β activity in progressive kidney diseases.

Cyr61 function is diverse and cell type specific [6], [8]. It is important to first identify the major cell type for Cyr61 expression in UUO kidneys. We demonstrated that Cyr61 was predominantly expressed in renal tubular epithelial cells, which might be related to its site of biological action. As a result, further *in vitro* experiments were performed on renal tubular epithelial cells. This distribution pattern was compatible with previous reports of renal Cyr61 expression [15]–[17] but was different from the pattern observed for CTGF in UUO kidneys [16], [18].

Animal studies have shown that UUO can elicit leukocyte (mainly macrophages) infiltration and lead to inflammation starting in the early phase [25], [41]. We showed that Cyr61 overexpression occurred early after UUO and was followed by MCP-1 upregulation. Based on this temporal relationship, it is tempting to speculate that Cyr61 may regulate MCP-1 expression after UUO. Results from *in vitro* experiments showed that treating renal tubular epithelial cells with Cyr61 can enhance MCP-1 expression. Without affecting TGF-β and CTGF expression, Cyr61 blockade using an anti-Cyr61 antibody in UUO mice attenuated renal MCP-1, CCR2, and F4/80 upregulation (Figure 6). Macrophage infiltration into the kidneys was also reduced by Cyr61 blockade during the early phase after UUO (Figure 6D). Collectively, these data suggest that Cyr61 may act as a proinflammatory modulator. This notion is compatible with an emerging body of evidence from relevant studies on macrophages, osteoblasts, fibroblasts, and endothelial cells [9], [13], [14].

Inflammation in UUO kidneys is thought to be an important factor for progressive interstitial fibrosis [29], [41]. A previous report showed that the blockade of the MCP-1 receptor CCR2 can attenuate renal fibrosis in the UUO model [42]. We demonstrated the Col 1-α1 and α-SMA expression were reduced at day 4 by anti-Cyr61 antibody treatment (Figure 7A–D). However, this beneficial effect could not persist during the later period of the disease to attenuate long-term fibrosis. Indeed, many previous studies using genetically engineered animals for UUO have also shown that the effect of knocking out specific molecules in the early phase of renal fibrosis diminished later when the pathology progressed [43]–[45]. There are some possible explanations to account for the incomplete efficacy of Cyr61 blockade. First, although CCN proteins have independent biological activity, they can also modify other molecules' signaling or serve as co-factors for others, depending on the particular biological system [5]. As a member of the CCN family, Cyr61 may work differentially depending on the changing microenvironment throughout the different stages of progressive renal fibrosis.

Second, macrophages comprise a heterogeneous population of cells and exhibit diverse roles between functionally distinct subpopulations [33]. They can be categorized into classically activated M1 macrophages (Th1 responses, proinflammatory phenotype) or alternatively activated M2 macrophage (Th2 responses, profibrotic or regulatory phenotype). Previous work has shown that during the course of UUO, peripheral blood monocytes are recruited to the obstructed kidneys, switched from M1-biased macrophages to M2-biased macrophages, and promote fibrogenesis [25], [34]. Because Cyr61 has been shown to induce Th1 responses in macrophages [14], its blockade may unbalance the macrophage polarization toward a profibrotic pattern. This hypothesis was supported by our quantification of renal *CCL17* and *CCL22* gene expression, which are two chemokines mainly derived from M2-biased macrophages [25], [32]–[34]. After anti-Cyr61 antibody treatment, both the *CCL17* to *F4/80* and *CCL22* to *F4/80* ratios increased in the UUO kidneys (Figure 6E and F).

Furthermore, previous studies have reported that Cyr61 can downregulate collagen expression and induce apoptosis in skin fibroblasts [9], [11], [12]. We also confirmed the inhibitory effect of Cyr61 on *Col 1-α1* and *Col 3-α1* expression in kidney fibroblasts (Supplement Figure S2). As a result, continuous Cyr61 blockade throughout the entire course of UUO would fail to suppress fibroblasts during the late period and might offset the antifibrotic effect noted in the early phase. Overall, the precise modulation of Cyr61 in a dose- and time-specific manner to improve disease outcome remains to be determined. Because a systemic knockout mutation in the Cyr61 gene is lethal [46], conditional knockout studies in particular cells types are warranted.

In conclusion, our results show that Cyr61 upregulation in response to chronic kidney injury played a proinflammatiory role in UUO disease progression. Further investigations into its mechanism of action in renal disease and its interplay with the microenvironment will be necessary to identify therapeutic targets in the Cyr61 signaling pathway.

## Supporting Information

Figure S1 **Neutralizing activity of the anti-Cyr61 antibody.** TSGH cell line is a human gastric carcinoma cell line expressing a high level of Cyr61 protein spontaneously. (Supplement Reference 1 and 2). The overexpressed Cyr61 of this cell line has been found to be associated with downstream upregulation of hypoxia-inducible factor (HIF)-1 α protein synthesis (Supplement Reference 2). To determine the neutralizing activity of the anti-Cyr61 antibody, TSGH cell was treated with 50 ng/mL of either control rabbit IgG or anti-Cyr61 antibody for 1 or 6 hours. Representative Western blots showed a significant reduction of HIF-1 α by anti-Cyr61 antibody treatment. Supplement Reference: 1. Lin MT, Zuon CY, Chang CC, Chen ST, Chen CP, et al. (2005) Cyr61 induces gastric cancer cell motility/invasion via activation of the integrin/nuclear factor-kappaB/cyclooxygenase-2 signaling pathway. Clin Cancer Res 11: 5809–5820. 2. Lin MT, Kuo IH, Chang CC, Chu CY, Chen HY, et al. (2008) Involvement of hypoxia-inducing factor-1alpha-dependent plasminogen activator inhibitor-1 up-regulation in Cyr61/CCN1-induced gastric cancer cell invasion. J Biol Chem 283: 15807–15815.
(TIF)

Figure S2 **Effect of Cyr61 on cultured renal fibroblast cells.** Cultured rat renal fibroblast cells (NRK-49F) were treated with recombinant Cyr61 protein for 3 days. Representative RT-PCR images of cDNA with (**A**) *Col 1-α1* and (**B**) *Col 3-α1* primers are shown in the upper. The graphs on the bottom show their relative gene expression normalized for *GAPDH*. *Col 1-α1* and *Col 3-α1* gene expression were suppressed 22 and 33%, respectively, by Cyr61 protein at a dose of 2 µg/mL. N = 3/group. The values are the mean+SD. *P<0.05 vs. control.
(TIF)
