# Supplementary figures and images for: Cysteine-Rich Protein 61 Plays a Proinflammatory Role in Obstructive Kidney Fibrosis
Source: PLoS One. 2013 Feb 15;8(2):e56481. doi: 10.1371/journal.pone.0056481 (PMC3574066; doi:10.1371/journal.pone.0056481)

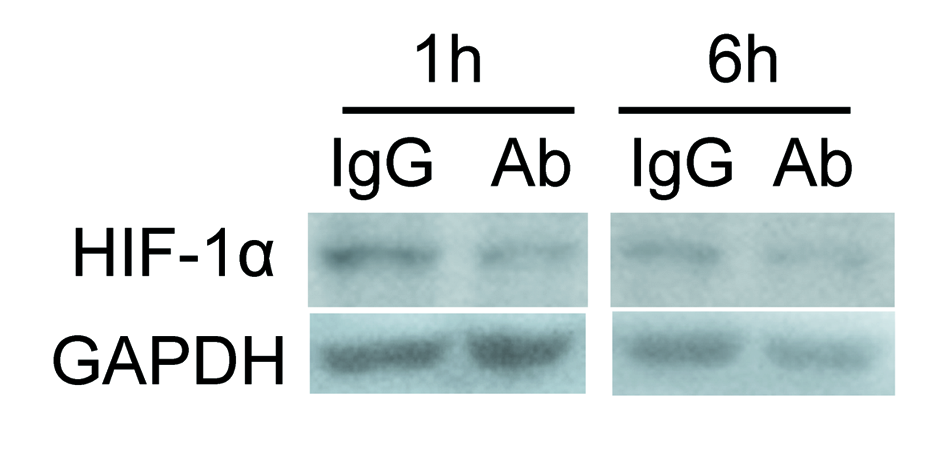

Supplement: Figure S1 — Neutralizing activity of the anti-Cyr61 antibody. TSGH cell line is a human gastric carcinoma cell line expressing a high level of Cyr61 protein spontaneously. (Supplement Reference 1 and 2). The overexpressed Cyr61 of this cell line has been found to be associated with downstream upregulation of hypoxia-inducible factor (HIF)-1 α protein synthesis (Supplement Reference 2). To determine the neutralizing activity of the anti-Cyr61 antibody, TSGH cell was treated with 50 ng/mL of either control rabbit IgG or anti-Cyr61 antibody for 1 or 6 hours. Representative Western blots showed a significant reduction of HIF-1 α by anti-Cyr61 antibody treatment. Supplement Reference: 1. Lin MT, Zuon CY, Chang CC, Chen ST, Chen CP, et al. (2005) Cyr61 induces gastric cancer cell motility/invasion via activation of the integrin/nuclear factor-kappaB/cyclooxygenase-2 signaling pathway. Clin Cancer Res 11: 5809–5820. 2. Lin MT, Kuo IH, Chang CC, Chu CY, Chen HY, et al. (2008) Involvement of hypoxia-inducing factor-1alpha-dependent plasminogen activator inhibitor-1 up-regulation in Cyr61/CCN1-induced gastric cancer cell invasion. J Biol Chem 283: 15807–15815. (TIF) [file pone.0056481.s001.tif]

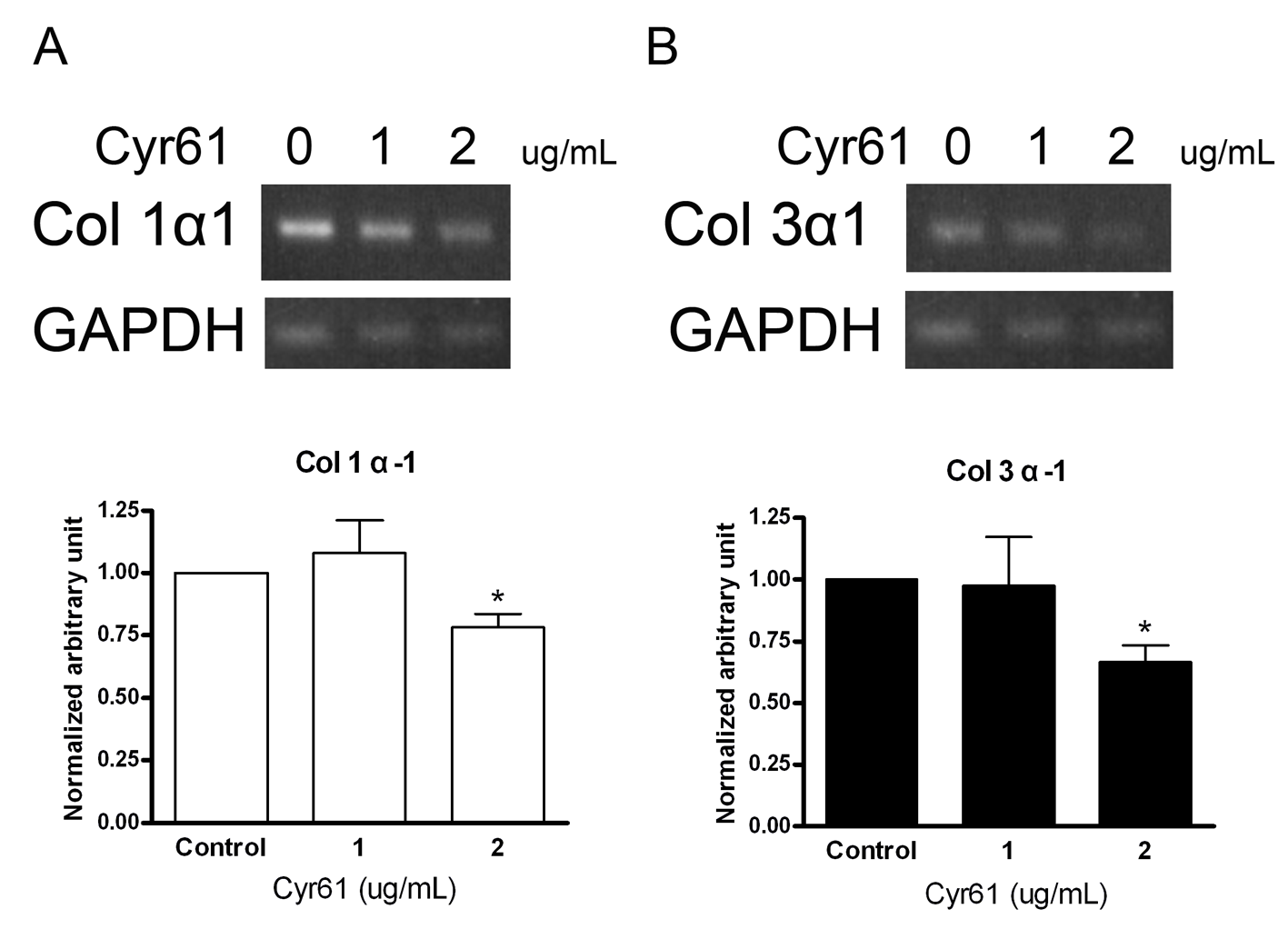

Supplement: Figure S2 — Effect of Cyr61 on cultured renal fibroblast cells. Cultured rat renal fibroblast cells (NRK-49F) were treated with recombinant Cyr61 protein for 3 days. Representative RT-PCR images of cDNA with (A) Col 1-α1 and (B) Col 3-α1 primers are shown in the upper. The graphs on the bottom show their relative gene expression normalized for GAPDH. Col 1-α1 and Col 3-α1 gene expression were suppressed 22 and 33%, respectively, by Cyr61 protein at a dose of 2 µg/mL. N = 3/group. The values are the mean+SD. *P<0.05 vs. control. (TIF) [file pone.0056481.s002.tif]
